# 5-Methylcytosine profiles in mouse transcriptomes suggest the randomness of m^5^C formation catalyzed by RNA methyltransferase

**DOI:** 10.1186/s13104-022-05968-7

**Published:** 2022-02-23

**Authors:** Junfeng Liu

**Affiliations:** 1 Key Laboratory of Genomic and Precision Medicine, Beijing Institute of Genomics, Chinese Academy of Sciences, Beijing, 100101 China; 2 China National Center for Bioinformation, Beijing, 100101 China; 3 Key Laboratory of Earth and Planetary Physics, Institute of Geology and Geophysics, Chinese Academy of Sciences, Beijing, 100029 China

**Keywords:** RNA 5-methylcytosine, Methylation rate, Methylation level

## Abstract

**Objective:**

5-Methylcytosine (m^5^C) is a type of chemical modification on the nucleotides and is widespread in both DNA and RNA. Although the DNA m^5^C has been extensively studied over the past years, the distribution and biological function of RNA m^5^C still remain to be elucidated. Here, I explored the profiles of RNA m^5^C in four mouse tissues by applying a RNA cytosine methylation data analysis tool to public mouse RNA m^5^C data.

**Results:**

I found that the methylation rates of cytosine were the same with the averages of methylation level at single-nucleotide level. Furthermore, I gave a mathematical formula to describe the observed relationship and analyzed it deeply. The sufficient necessary condition for the given formula suggests that the methylation levels at most m^5^C sites are the same in four mouse tissues. Therefore, I proposed a hypothesis that the m^5^C formation catalyzed by RNA methyltransferase is random and with the same probability at most m^5^C sites, which is the methylation rate of cytosine. My hypothesis can be used to explain the observed profiles of RNA m^5^C in four mouse tissues and will be benefit to future studies of the distribution and biological function of RNA m^5^C in mammals.

**Supplementary Information:**

The online version contains supplementary material available at 10.1186/s13104-022-05968-7.

## Introduction

5-Methylcytosine (m^5^C) is a type of chemical modification on the nucleotides and is widespread in both DNA and RNA. DNA m^5^C is a common epigenetic modification which is crucial for diverse biological processes, including gene silencing, imprinting and X chromosome inactivation [1], and the aberrant m^5^C has been associated with multiple diseases, such as Alzheimer’s disease and cancer [2, 3]. Although the DNA m^5^C has been extensively studied over the past years, the distribution and biological function of RNA m^5^C still remain to be elucidated [4]. However, some current studies showed that RNA m^5^C and m^5^C RNA methyltransferases play important roles in the development and pathogenesis of cancer [5–7].

A combination of bisulfite treatment of RNA and followed by PCR-based amplification of cDNA and DNA sequencing is an important approach to detect the m^5^C sites [8]. The high-throughput sequencing of RNA treated with bisulfite (RNA-BisSeq) can be used to profile RNA m^5^C at single-nucleotide resolution. Currently, some tools have been developed to analyze RNA-BisSeq, such as meRanTK [9] and Episo [10]. By analyzing RNA-BisSeq data of mouse embryonic stem cells and murine brain, Amort et al. [11] observe a pronounced accumulation of m^5^C sites in the vicinity of the translational start codon, depletion in coding sequences, and mixed patterns of enrichment in the 3’UTR. By analyzing human and mouse RNA-BisSeq data, Yang et al. [12] reveal that m^5^C modification is enriched in CG-rich regions and in regions immediately downstream of translation initiation sites and Liu et al. [10] find that the RNA m^5^C is not evenly distributed among the transcript isoforms at isoform level. However, only partial m^5^C sites were analyzed in the above studies. The methylation level of candidate cytosine positions should be no less than 0.2 [8] and 0.1 [10, 12], respectively.

In this study, I mapped m^5^C globally in human HeLa cells and multiple mouse tissues using RNA-BisSeq data and deeply analyzed the relationship among global methylation rate, methylation level at single-nucleotide resolution and methylation level at gene resolution. Collectively, the results suggest that the m^5^C formation catalyzed by RNA methyltransferase is random.

## Main text

### Methods

#### Data sources

I downloaded RNA-BisSeq data of human and mouse from the BIG Data Center under accession number PRJCA000315 [13]. Furthermore, I downloaded reference genome and transcriptome of human (version GRCh37) and mouse (version GRCm38) from the Ensemble database [14].

#### RNA-BisSeq bioinformatics analysis

The alignment procedure was performed by using Episo [10], which maps RNA-BisSeq reads to the reference genome and reference transcriptome. Episo can convert the m^5^C sites in transcriptome and junction sequences to corresponding genome locus. I define the methylation rate and methylation level as follows. The methylation rate of cytosine is the proportion of unconverted cytosine in all examined RNA-BisSeq data. The methylation rate of reads is the proportion of the reads with methylation in all examined RNA-BisSeq reads. The methylation level at single-nucleotide level is defined as *i*/(*i* + *j*), where *i* denotes the number of reads with methylation at the given m^5^C site, and *j* denotes the number of reads lack of methylation at the given m^5^C site. The methylation level at gene level is defined as ${{R_{m,g} } \mathord{\left/ {\vphantom {{R_{m,g} } {R_{g} }}} \right. \kern-\nulldelimiterspace} {R_{g} }}$, where $R_{m,g}$ denotes the number of reads that carry at least one methylated cytosine site from the given gene, and $R_{g}$ denotes the number of reads that come from the given gene. I only analyzed the sites with coverage depth > 30.

#### Simulation

I simulated an RNA-Seq experiment using the FluxSimulator with default parameters [15], which is a freely available software package that models whole-transcriptome sequencing experiments with the Illumina Genome Analyzer. The software works by first randomly assigning expression values to the transcripts provided by user, constructing an amplified, size-selected library, and then sequencing it. Human transcripts assembled by Cufflinks [16] according to the experimental data were supplied to the FluxSimulator. FluxSimulator then randomly assigned expression levels to 40,205 transcripts and produced paired-end RNA-Seq reads that the length is 101-bp and the numbers are 23 million. Then, I simulated the bisulfite treatment using the Bisulfitefq, which comes from the package Episo. The probability of methylation at each m^5^C site is 0.001.

### Results

#### 5-Methylcytosine profiles in mouse transcriptomes

To explore the profiles of m^5^C in mouse transcriptomes, I applied Episo [10] to published RNA-BisSeq data in mouse (liver, kidney, heart and brain). According to the mapping results from Episo, I computed the methylation rate of cytosine and the methylation rate of reads in the published RNA-BisSeq data (Table 1). In four mouse tissues (liver, kidney, heart and brain), the methylation rates of cytosine were all 0.001 and the methylation rates of reads were 0.034, 0.029, 0.035 and 0.038 respectively. When computing the methylation level at single-nucleotide level, I analyzed the sites with coverage depth > 30. In four mouse tissues, the averages of methylation level at single-nucleotide level were all 0.001. The averages of methylation level at gene level in four mouse tissues (liver, kidney, heart and brain) were 0.034, 0.030, 0.030, and 0.039 respectively. Moreover, the methylation level at gene level in brain is significantly higher than that in the other three tissues (Additional file 2: Figure S1). This suggested that the RNA m^5^C at gene level tends to be tissue-specific. Liu et al. [10] also showed the similar conclusion that the RNA m^5^C at isoform level was tissue specific. Intriguingly, the methylation rates of cytosine in four mouse tissues were all the same and were the same with the averages of methylation level at single-nucleotide level. This indicated that the methylation rates of cytosine were conserved across four mouse tissues and there was some relationship between methylation rate of cytosine and the methylation level at single-nucleotide level. In addition, the methylation rates of reads were also close to the averages of methylation level at gene level in four mouse tissues. This showed that the same relationship also existed between the methylation rate of read and the methylation level at gene level.

**Table 1:** 5-Methylcytosine profiles in mouse transcriptomes

Tissue	Methylation rate_A	Methylation level_A	Methylation rate_B	Methylation level_B
Liver	0.001	0.001	0.034	0.034
Kidney	0.001	0.001	0.029	0.030
Heart	0.001	0.001	0.035	0.030
Brain	0.001	0.001	0.038	0.039

Methylation rate_A means the methylation rate of cytosine; methylation level_A means the average of methylation level at single-nucleotide level; methylation rate_B means the methylation rate of reads; methylation level_B means the average of methylation level at gene level. For computing the methylation level at single-nucleotide level, I analyzed the sites with coverage depth > 30

#### A hypothesis

The profiles of m^5^C in mouse transcriptomes showed that the methylation rates of cytosine were the same with the averages of methylation level at single-nucleotide level in four mouse tissues. It can be described as the following formula:

1 $$\left(\frac{{a}_{1}}{{b}_{1}}+\frac{{a}_{2}}{{b}_{2}}+\cdots +\frac{{a}_{n}}{{b}_{n}}\right)/n\approx \frac{{a}_{1}+{a}_{2}+\cdots +{a}_{n}}{{b}_{1}+{b}_{2}+\cdots +{b}_{n}}$$

where $n$ denotes the number of m^5^C sites; ${a}_{i}$ denotes the number of reads with methylation at the ${i}_{th}$ m^5^C site; ${b}_{i}$ denotes the number of reads at the ${i}_{th}$ m^5^C site. The left of formula (1) means the average of methylation level at single-nucleotide level and the right of formula (1) means the methylation rate of cytosine. In mathematics, the sufficient necessary condition for the formula (1) is $\frac{{a}_{1}}{{b}_{1}}=\frac{{a}_{2}}{{b}_{2}}=\cdots =\frac{{a}_{n}}{{b}_{n}}$ (Additional file 1). Because $\frac{{a}_{i}}{{b}_{i}}$ is the methylation level at the ${i}_{th}$ m^5^C site, the sufficient necessary condition for the formula (1) suggests that the methylation levels at most m^5^C sites are the same. How to explain the suggestion from the sufficient necessary condition for the formula (1)? If the m^5^C formation catalyzed by RNA methyltransferase is random and with the same probability at most m^5^C sites, the methylation level at the ${i}_{th}$ m^5^C site is the probability of methylation at the ${i}_{th}$ m^5^C site according to law of large numbers when ${b}_{i}$ is sufficiently large, and the suggestion from the sufficient necessary condition for the formula (1) can be explained. Therefore, I proposed a hypothesis that the m^5^C formation catalyzed by RNA methyltransferase is random and with the same probability at most m^5^C sites, which is the methylation rate of cytosine. According to my hypothesis, the average of methylation level at gene level should be close to the methylation rate of reads (Table 1). Furthermore, I explored the profile of m^5^C in human HeLa cells. The methylation rate of cytosine was still the same with the average of methylation level at single-nucleotide level, and was equal to the methylation rates of cytosine in four mouse tissues.

### Discussion

In my hypothesis, the probability at most m^5^C sites can be obtained by computing the methylation rate of cytosine. In order to in silico examine the conclusion, I simulated RNA-BisSeq data, in which the m^5^C formation was random and the probability of methylation at each m^5^C site was 0.001. Then, I applied Episo to the simulated RNA-BisSeq data and computed the methylation rate of cytosine. The results showed that the methylation rate of cytosine is equal to the probability given by simulating (Additional file 2: Table S1). Furthermore, the average of methylation level at single-nucleotide level was also 0.001 and the methylation rate of reads was close to the average of methylation level at gene level. The simulation results that are obeyed to the hypothesis are consistent with the observed results in four mouse tissues.

There are several tools for RNA-BisSeq data analysis, such as meRanTK [9], BS-RNA [17], BisRNA [18], BisAMP [19], and Episo [10]. Although meRanTK is the first available tool for RNA-BisSeq data analysis [20, 21] and has been performed to present the picture of RNA m^5^C in the mouse embryonic stem cells and brain [11], I used Episo to analyze RNA-BisSeq data in this study. There are two reasons for selecting Episo. The first reason is the high accuracy of Episo. Liu et al. [10] assessed the performance of Episo with a set of in silico experiments and the results showed that Episo accurately estimated the methylation rates, as well as the average differences between the estimated and simulated methylation levels were nearly zero. Moreover, Liu et al. [10] performed experimental assessment of Episo with MeRIP followed by qPCR and the results showed that the experimental observed methylation levels have the same trends with the estimates. The second reason is that Episo has the higher mapping rates compared to meRanTK. Under three methylation rates tested, the mapping rates of Episo and meRanTK were 86.6% and 80.72% respectively [10].

Current studies mainly focus on the distribution of m^5^C sites with high methylation level. However, the mechanism of distribution of m^5^C sites with high methylation level is not clear. My hypothesis may be helpful to explore the mechanism. According my hypothesis, the following three factors may cause the high methylation level at some m^5^C sites. The first factor is that the coverage depth is low. If the coverage depth at one m^5^C site is 10, the methylation level is at least 0.1 when the site was catalyzed by RNA methyltransferase. This indicates that it may be negative correlation between the RNA m^5^C level at single-nucleotide level and the coverage depth. Liu et al. [10] found a weak negative correlation between the RNA m^5^C level at isoform level and the isoform expression in four mouse tissues (liver, kidney, heart and brain). The second factor is the CG-rich environments. Yang et al. [12] found that m^5^C sites with high methylation level were embedded in CG-rich environments. The third factor is the aberrant RNA methyltransferase that can cause the high probability of methylation. David et al. [22] found that the overexpression of the RNA m^5^C methyltransferase TRM4B specifically increased the methylation level. Therefore, the mechanism of distribution of m^5^C sites with high methylation rate should be related to the gene expression, CG-rich environments and the aberrant expression of or mutations in RNA methyltransferase.

In summary, I explored the profiles of m^5^C in mouse transcriptomes by computing the methylation rate and the methylation level. I found that the methylation rates of cytosine were the same with the averages of methylation level at single-nucleotide level in four mouse tissues. Furtherly, I explored the profile of m^5^C in human HeLa cells and observed the same relationship between the methylation rates of cytosine and the average of methylation level at single-nucleotide level. I gave a mathematic formula to describe the above relationship and analyzed it deeply. The sufficient necessary condition for the given formula suggests that the methylation levels at most m^5^C sites are the same in four mouse tissues and human HeLa cells. In order to explain the above observation, I proposed a hypothesis that the m^5^C formation catalyzed by RNA methyltransferase is random and with the same probability at most m^5^C sites, which is the methylation rate of cytosine. Finally, I simulated RNA-BisSeq data with the randomness of m^5^C formation catalyzed by RNA methyltransferase to test whether the probability at most m^5^C sites can be obtained by computing the methylation rate of cytosine. I hope my hypothesis will facilitate additional research required to understand the distribution and biological function of RNA m^5^C in mammals.

## Limitations

Though the mathematical proof shows that the methylation levels at most m^5^C sites are the same, additional work is needed to prove that methylation levels at different m^5^C sites are not significantly different as well as to consider the measure of uncertainties. The mechanism of distribution of m^5^C sites with high methylation level has not been elaborated.

## Supplementary Information

**Additional file 1. **Proposition. Detailed mathematical proof of the proposition.

**Additional file 2: Table S1.** 5-Methylcytosine profiles in simulated RNA-BisSeq data. Figure S1. The distribution of methylation level at gene level.

## Data Availability

All data generated or analyzed during this study are included in this published article and its supplementary information files. RNA-BisSeq data of human and mouse are from the BIG Data Center (https://ngdc.cncb.ac.cn) under accession number PRJCA000315. The reference genome and transcriptome of human (version GRCh37) and mouse (version GRCm38) are from the Ensemble database (https://asia.ensembl.org/index.html). The public access to the databases is open.
