# Supplementary material for: 5-Methylcytosine profiles in mouse transcriptomes suggest the randomness of m5C formation catalyzed by RNA methyltransferase
Source: BMC Res Notes. 2022 Feb 23;15:81. doi: 10.1186/s13104-022-05968-7 (PMC8867762; doi:10.1186/s13104-022-05968-7)
Supplement: Supplementary file 1 — Additional file 1. Proposition. Detailed mathematical proof of the proposition. [file 13104_2022_5968_MOESM1_ESM.pdf]

Supplementary Material

5-methylcytosine profiles in mouse transcriptomes suggest the randomness of m^{5}C formation catalyzed by RNA methyltransferase

Proposition

If $\left(\frac{a_1}{b_1}+\frac{a_2}{b_2}+\cdots+\frac{a_n}{b_n}\right)/n=\frac{a_1+a_2+\cdots+a_n}{b_1+b_2+\cdots+b_n}$, where $a_i \geq 0, b_i > 0, a_i \leq b_i \;\; i = 1,2,\cdots,n$, then the sufficient necessary condition is $\frac{a_1}{b_1}=\frac{a_2}{b_2}=\cdots=\frac{a_n}{b_n}$.

Proof:

Sufficient: Because $\frac{a_1}{b_1}=\frac{a_2}{b_2}=\cdots=\frac{a_n}{b_n}$, let $\frac{a_i}{b_i}=k \;\; i=1,2,\cdots,n$. Therefore, $\left(\frac{a_1}{b_1}+\frac{a_2}{b_2}+\cdots+\frac{a_n}{b_n}\right)/n=k$ and $a_i=k\times b_i \;\; i=1,2,\cdots,n$. I can obtain $\frac{a_1+a_2+\cdots+a_n}{b_1+b_2+\cdots+b_n}=\frac{k\times(b_1+b_2+\cdots+b_n)}{b_1+b_2+\cdots+b_n}=k$. So, $\left(\frac{a_1}{b_1}+\frac{a_2}{b_2}+\cdots+\frac{a_n}{b_n}\right)/n=\frac{a_1+a_2+\cdots+a_n}{b_1+b_2+\cdots+b_n}$. The sufficient proof is completed.

Necessary: Let $k=min\left\{\frac{a_1}{b_1},\frac{a_2}{b_2},\cdots,\frac{a_n}{b_n}\right\}=\frac{a_j}{b_j}$, then $\frac{a_1}{b_1}=k+\varepsilon_1$, $\frac{a_2}{b_2}=k+\varepsilon_2,\cdots,\frac{a_n}{b_n}=k+\varepsilon_n$, $\varepsilon_1,\cdots,\varepsilon_n \geq 0$ 且$\varepsilon_j=0$. Because $\left(\frac{a_1}{b_1}+\frac{a_2}{b_2}+\cdots+\frac{a_n}{b_n}\right)/n=\frac{a_1+a_2+\cdots+a_n}{b_1+b_2+\cdots+b_n}$, I can obtain $\sum_{i=1}^{n} m_i=0$ and $\sum_{i=1}^{n} m_i\varepsilon_i=0,$ $where$ $m_i=\frac{(b_1+b_2+\cdots+b_n)-nb_i}{n(b_1+b_2+\cdots+b_n)}$ $i=1,2,3,\cdots,n$. Furthermore, $\sum_{i=1}^{j-1}[(b_1+b_2+\cdots+b_n)-nb_i]\,\varepsilon_i+\sum_{i=j+1}^{n}[(b_1+b_2+\cdots+b_n)-nb_i]\,\varepsilon_i=0$. When $b_j > max\{(n-1)b_i,\ i\neq j\}$, I can get $\varepsilon_i=0, i\neq j$. So, If $\sum_{i=1}^{n} m_i\varepsilon_i=0$ for arbitrary $\{b_i\}$, then $\frac{a_1}{b_1}=\frac{a_2}{b_2}=\cdots=\frac{a_n}{b_n}$. The necessary proof is completed.
