# Supplementary material for: 5-Methylcytosine profiles in mouse transcriptomes suggest the randomness of m5C formation catalyzed by RNA methyltransferase
Source: BMC Res Notes. 2022 Feb 23;15:81. doi: 10.1186/s13104-022-05968-7 (PMC8867762; doi:10.1186/s13104-022-05968-7)
Supplement: Supplementary file 2 — Additional file 2: Table S1. 5-Methylcytosine profiles in simulated RNA-BisSeq data. Figure S1. The distribution of methylation level at gene level. [file 13104_2022_5968_MOESM2_ESM.pdf]

Supplementary Material

5-methylcytosine profiles in mouse transcriptomes suggest the randomness of m^{5}C formation catalyzed by RNA methyltransferase

Table S1. 5-methylcytosine profiles in simulated RNA-BisSeq data

methylation rate_A	methylation level_A	methylation rate_B	methylation level_B
0.001	0.001	0.046	0.047

Note. methylation rate_A means the methylation rate of cytosine; methylation level_A means the average of methylation level at single-nucleotide level; methylation rate_B means the methylation rate of reads; methylation level_B means the average of methylation level at gene level. For the simulated RNA-BisSeq data, the m^{5}C formation was random and the probability of methylation at each m^{5}C site was 0.001.

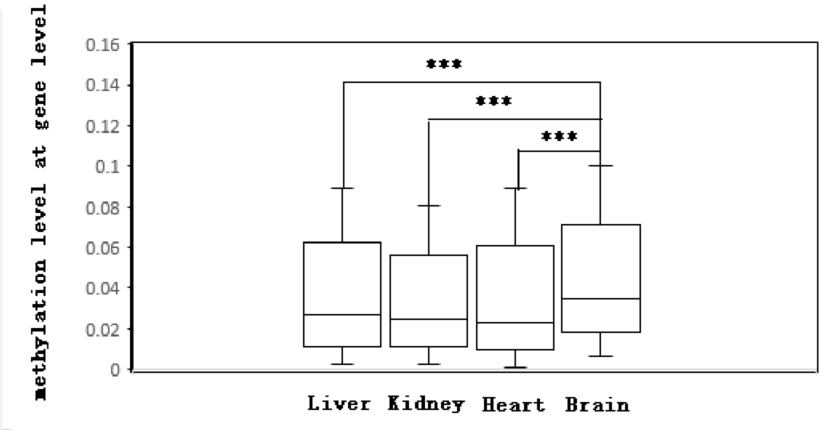

Fig. S1 The distribution of methylation level at gene level. In four mouse tissues, the methylation level at gene level in brain is significantly higher than that in the other three tissues. The '***' indicates that significant difference of *P*-value < 0.001.
